# The role of combined ultrasonic BI-RADS and ultrasound elastography in discriminating between benign and malignant breast nodules

**DOI:** 10.1097/MD.0000000000047117

**Published:** 2026-01-16

**Authors:** Lin Lu, Limin Zhu, Jing Li, Pei Zou

**Affiliations:** a The Ultrasound Diagnosis Department, Shaanxi Nuclear Industry No. 215 Hospital, Xianyang City, Shanxi Province, China.

**Keywords:** breast imaging-reporting and data system, breast nodules, diagnosis, ultrasonic, ultrasound elastography

## Abstract

This research is designed to examine how well the combination of ultrasonic breast imaging-reporting and data system (BI-RADS) classification and ultrasound elastography (UE) works in discriminating between benign and malignant breast nodules (BNs). It also seeks to confirm the clinical applicability of this combined approach in diagnostically challenging cases involving dense breast tissue and small BNs. We retrospectively reviewed 280 patients with BNs (142 assessed by BI-RADS alone, and 138 evaluated with the combined approach) between March 2023 and February 2025. Using pathological biopsy as the gold standard, the diagnostic performance – including sensitivity, specificity, accuracy, and Kappa value – of the 2 groups was compared. Additional subgroup assessments focused on dense breast tissues (American College of Radiology categories C/D) and small nodules (≤5mm). The sensitivity, specificity and accuracy of BI-RADS combined with UE in the diagnosis of malignant BN were 91.30%, 86.96% and 89.86%, respectively (Kappa = 0.886), which was better than that of BI-RADS alone. In addition, the diagnostic sensitivity of BI-RADS combined with UE for dense nodules and small nodules was also higher than that of BI-RADS alone (*P* <.05). The combined use of ultrasonic BI-RADS and UE enhances diagnostic accuracy by concurrently evaluating morphological features and tissue stiffness, offering improved differentiation between benign and malignant BNs.

## 1. Introduction

Breast cancer (BC) remains the most frequently diagnosed cancer in women worldwide. The World Health Organization reported in 2023 that more than 2.3 million new cases are identified, with around 680,000 fatalities attributed to the disease.^[1]^ In China, the incidence rate of BC, while still lower than that in Western countries, has been rising significantly. Moreover, cases are increasingly occurring among younger women, making it a growing public health concern.^[2]^ The prognosis of BC varies considerably by stage; early detection is associated with a 5-year survival rate of over 90%, while late-stage diagnosis drops to under 30%. Thus, correctly classifying breast nodules (BNs) as benign or malignant at an early point is vital to achieving better patient outcomes.^[3]^ In 2003, the American College of Radiology introduced the Breast Imaging-Reporting and Data System (BI-RADS). This system uses standardized ultrasound terminology – such as echo, boundary, and blood flow – and a scoring system (categories 1–6), which has greatly improved the consistency of imaging reports and their value in guiding clinical practice.^[4]^ Nevertheless, BI-RADS classification has its shortcomings. It often misjudges certain nodules that have clear boundaries but uneven internal echoes, like fibroadenomas and atypical hyperplasia. Moreover, it fails to take into account tissue stiffness, a key biological characteristic.^[5]^

Ultrasound elastography (UE) is an advanced technology developed in recent years. It gauges tissue stiffness by quantifying either the tissue’s ability to deform or the speed at which shear waves propagate through the tissue.^[6]^ Research suggests that UE significantly enhances the differentiation of benign and malignant BNs when complementing the BI-RADS framework.^[7]^ This integrated approach provides valuable insights for the early detection of BC and aids in developing tailored management strategies. Nevertheless, many prior investigations are limited in scale and nodule diversity, having predominantly examined solid nodules.^[8]^ Furthermore, limited subgroup analyses targeting different populations (e.g., women with dense versus non-dense breast tissue) hinder a clear assessment of the combined method’s general applicability.

Therefore, this study intends to conduct an in-depth analysis of the diagnostic application of the combination of BI-RADS and UE in identifying benign and malignant BNs. Validation of this combined protocol as a superior adjunct tool would provide a more robust basis for clinical decision-making. On one hand, it could reduce unnecessary biopsies, easing the psychological stress on patients and cutting down on medical costs. On the other hand, it could enhance the detection of high-risk BNs, facilitating timely surgical intervention or more vigilant monitoring to improve overall patient outcomes. Furthermore, establishing a standardized joint diagnostic protocol will promote the homogenization of ultrasound practices. This initiative will enhance the ability of primary care hospitals to diagnose and treat breast diseases, thereby providing evidence-based support for refining strategies for early-stage BC screening and diagnosis.

## 2. Materials and methods

### 2.1. Research participants

This study was approved by the Ethics Committee of Hai’an Hospital of Shaanxi Nuclear Industry No. 215 Hospital. This was a retrospective analysis, focusing on patients with confirmed BNs treated at our institution between March 2023 and February 2025. To establish the required sample size, a power analysis was performed via G*Power. Based on values reported in an earlier study^[9]^–including a 2-tailed alpha level of 0.05, statistical power of 80%, and an anticipated between-group difference of 10%–the analysis indicated that each group should include at least 120 participants. Taking into account a potential 10% dropout rate, the final number of cases for the study was set at 134 per group. Following the application of patient selection criteria, 142 patients assessed solely with BI-RADS (the BI-RADS group) and 138 patients evaluated with both BI-RADS and UE (the combined group) were enrolled. The study protocol received approval from the hospital’s ethics committee, and informed consent was obtained from every participant.

### 2.2. Patient selection criteria

Inclusion criteria: Female patients aged 18 to 75 years; presence of a single BN (8–30 mm in diameter) detected through palpation or conventional ultrasound; first-time visitors or no history of breast surgery, radiotherapy, or endocrine treatment in the preceding 6 months; voluntary agreement to participate in the study with the provision of informed consent.

Exclusion criteria: Pregnant or lactating status; acute mastitis or breast abscess (accompanied by redness, swelling, heat, and pain); nodules with coarse calcification (ACR BI-RADS category 4 or above) or invasion into the skin/chest wall; previous breast prosthesis implantation, breast augmentation, or other plastic surgeries; severe cardiac, hepatic, renal insufficiency, or psychiatric conditions that could compromise examination compliance; poor-quality ultrasound images (e.g., nodules obscured by gas or bones, or severe image artifacts) precluding a conclusive BI-RADS evaluation or UE.

### 2.3. BI-RADS examination

A GE Voluson E10 or Philips EPIQ 7 color Doppler ultrasound machine, equipped with a high-frequency linear array probe (frequency range: 7–14 MHz), was used for the examination. Patients were placed supine, with their arms raised to fully expose the breasts; when necessary, supplementary scanning was performed with the patient in a lateral position. Independent assessment was performed by 2 senior sonographers (≥5 years of experience) using the following indicators: nodule size (length × width × height), morphology (regular/irregular), boundary (well-/ill-defined), echogenicity (hypoechoic/isoechoic/hyperechoic/mixed), internal structure (solid/cystic/mixed), vascularity grade (0–3 per Adler classification), and calcification (none/micro/coarse). Discrepancies were resolved by a third reader. Final classification followed the American College of Radiology BI-RADS guidelines, with category 4B and above considered suspicious for malignancy, warranting further investigation.

### 2.4. UE examination

In strain elastography mode, the probe was lightly applied to the nodule surface. After image stabilization, the capture was frozen using a standardized compression depth of 2 mm. A circular region of interest (ROI) with a 2-mm diameter was delineated to fully encompass the nodule. For nodules larger than 20 mm, the ROI encompassed the central portion as well as the periphery. The compression depth and force were kept constant to obtain elastographic images of the nodule, which were then scored. The scoring criteria were as follows: 1 point, uniform green; 2 points, predominantly green with minimal blue; 3 points, mixed blue and green; 4 points, mostly blue with limited green; 5 points, entirely blue. A score ≥ 4 is considered characteristic of malignant lesions.

### 2.5. Pathological examination

For BNs categorized as BI-RADS 4 or above, or when patients explicitly requested biopsy, core needle biopsy or surgical resection biopsy was performed. Nodes classified as BI-RADS 1 to 3 were monitored clinically if the patient declined biopsy (follow-up ultrasound was performed after 6 months; biopsies were conducted if growth or malignant features were observed). The final pathological results were independently diagnosed by 2 pathologists. Lesions were classified as malignant if they were invasive carcinoma or carcinoma in situ, and as benign if they were fibroadenoma, adenosis, or cysts.

### 2.6. Quality control

All ultrasound physicians received unified training on operational procedures, including the use of BI-RADS terminology and elastography parameter settings. They were only allowed to participate in the study after passing a qualification assessment. Dynamic images, including both conventional ultrasound and UE images, were stored during each examination. Two physicians reviewed these images without knowing the pathological results (blind review). Images that did not meet the quality standards – such as those with mispositioned ROIs in UE or inadequate nodule depiction in conventional ultrasound – were discarded. Additionally, a dual-entry procedure was employed for database input. The consistency between entries was regularly assessed, requiring a Kappa coefficient exceeding 0.8.

### 2.7. Statistical analysis

Data analysis was performed with SPSS 34.0 (Chicago). The assessment of normality for measurement data was conducted via the Shapiro–Wilk test. For data adhering to a normal distribution, values are presented as ($\bar{\chi }\pm s$), and group comparisons were made with an independent-samples *t*-test. For data that violated the assumption of normality, presented as median (IQR), the nonparametric Mann–Whitney *U* test was utilized for analysis. Count data, expressed as (n [%]), were subjected to chi-square testing. With the pathological biopsy results as the gold standard, the Kappa test was used to assess diagnostic performance. A threshold of *P* <.05 was applied to establish statistical significance for inter-group comparisons.

## 3. Results

### 3.1. Baseline data comparison

We first conducted an inter-group comparison of patients’ clinical data, confirming their comparability as no marked differences were found in age, family medical history, body mass index, menstrual status, etc (*P* >.05, Table 1).

**Table 1:** Comparison of clinical data.

	BI-RADS (n = 142)	BI-RADS combined with UE (n = 138)	*t* (or χ^2^)	*P*
Age (year old)	53.01 ± 9.22	52.27 ± 9.66	0.655	.513
BMI (kg/m^2^)	23.26 ± 1.97	23.05 ± 2.17	0.851	.396
Menstrual status				
Menopause	75 (52.82)	68 (49.28)	0.351	.553
Not in menopause	67 (47.18)	70 (50.72)		
Family history of BC				
Have	8 (5.63)	12 (8.70)	0.989	.320
None	134 (94.37)	126 (91.30)		
Smoking				
Yes	42 (29.58)	35 (25.36)	0.624	.430
No	100 (70.42)	103 (74.64)		
Drinking				
Yes	20 (14.08)	24 (17.39)	0.578	.447
No	122 (85.92)	114 (82.61)		
CA15-3 (U/mL)	29.25 ± 8.06	28.21 ± 9.92	0.962	.337
CA27.29 (U/mL)	23.71 ± 12.53	22.81 ± 9.94	0.670	.503
CEA (ng/mL)	4.41 ± 2.02	4.69 ± 2.19	1.124	.262

BC = breast cancer, BI-RADS = breast imaging-reporting and data system, BMI = body mass index, BNs = breast nodules, CEA = carcinoembryonic antigen, CI = confidence interval.

### 3.2. Pathological biopsy results

**Figure 1.**
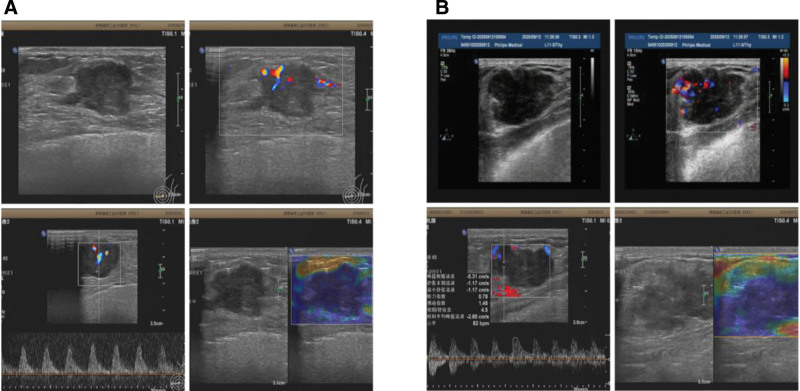
Imaging findings for 2 subjects. (A) Classified as BI-RADS 5 based on examination results. (B) Classified as BI-RADS 4b based on examination results. BI-RADS = breast imaging-reporting and data system.

Pathological biopsy confirmed 196 malignant BNs, with 104 in the BI-RADS group and 92 in the combined group. As shown in Figure 1, we present examination results for 2 research subjects. (A) is a 55-year-old female. Two-dimensional ultrasound revealed a left breast mass (approximately 17.1 × 14.7 mm in size) with poorly defined borders, irregular shape, and visible lobulation. Blood flow examination reveals large vessels. Spectral Doppler shows high-velocity, high-resistance arterial blood flow spectrum (vs 15.2 cm/s, vs 2.43 cm/s, RI 0.84), classified as BI-RADS 5. Elastography images display uniformly blue. (B) is a 33-year-old female. Two-dimensional ultrasound revealed a mass at the 2 o’clock position in the right breast (approximately 31.2 × 19.1 mm in size), with poorly defined borders and irregular shape, classified as BI-RADS 4b.

### 3.3. Diagnostic efficacy of BI-RADS

When assessed against pathological biopsy results, the BI-RADS classification achieved 84 true positives and 32 true negatives in diagnosing benign and malignant BNs. It showed 80.77% sensitivity, 84.21% specificity, and 81.69% accuracy for malignancy diagnosis, with a Kappa coefficient of 0.814 (*P* <.001, Table 2), which denotes reasonable diagnostic reliability.

**Table 2:** Efficacy of BI-RADS in the diagnosis of BNs.

n = 142		Gold standard		
		(+)	(−)	Total
BI-RADS	(+)	84	6	90
	(−)	20	32	52
	Total	104	38	
95% CI	0.642–0.954			
Kappa	0.834			
*P*	<.001			

BI-RADS = breast imaging-reporting and data system, BNs = breast nodules, CI = confidence interval.

### 3.4. Diagnostic efficacy of BI-RADS combined with UE

When used together, BI-RADS and UE yielded 84 true positive and 40 true negative cases. The diagnostic performance included a sensitivity of 91.30%, specificity of 86.96%, and accuracy of 89.86%. The Kappa coefficient comparing this combined method to pathological findings was 0.886 (*P* <.001, Table 3), denoting substantial agreement.

**Table 3:** Efficacy of BI-RADS combined with UE in the diagnosis of BNs.

n = 138		Gold standard		
		(+)	(−)	Total
BI-RADS combined with UE	(+)	84	6	90
	(−)	8	40	48
	Total	92	46	
95% CI	0.721–0.973			
Kappa	0.886			
*P*	<.001			

BI-RADS = breast imaging-reporting and data system, BNs = breast nodules, CI = confidence interval, UE = ultrasound elastography.

### 3.5. Subgroup analysis

Dense breast tissue can limit ultrasound wave penetration, influencing the clarity of nodule imaging and the precision of elastographic assessments.^[10]^ We therefore performed a subgroup analysis involving patients with dense breasts and BNs. Of the 102 subjects in this category, 54 were in the BI-RADS group and 48 were in the combined group. The comparison results showed that the diagnostic sensitivity of BI-RADS combined with UE group for dense BN was higher than that of BI-RADS group (*P* = .036). Secondly, small nodules (<5mm) may cause unstable measurement of elastic parameters due to their small size.^[11]^ Therefore, we performed a subgroup analysis again for the 95 patients with small nodules in this study. Statistical analysis showed that the diagnostic sensitivity of BI-RADS group for small nodules was 88.89%, which was also higher than that of BI-RADS group (*P* = .040, Table 4).

**Table 4:** Diagnostic performance of BI-RADS and BI-RADS combined with UE for dense BNs and small nodules.

Dense BNs				
	n	Sensitivity	χ^2^	*P*
BI-RADS	54	38 (70.37)	4.408	.036
BI-RADS combined with UE	48	42 (87.50)		

Small nodules				
	n	Sensitivity	χ^2^	*P*
BI-RADS	50	36 (72.00)	4.222	.040
BI-RADS combined with UE	45	40 (88.89)		

BI-RADS = breast imaging-reporting and data system, BNs = breast nodules, UE = ultrasound elastography.

## 4. Discussion

This study conducted a comparative analysis to evaluate the performance of BI-RADS classification alone and in conjunction with UE for distinguishing between benign and malignant BNs. The combined method showed higher diagnostic consistency with pathology results (Kappa = 0.846) than BI-RADS used independently. Further subgroup analyses highlighted its utility in evaluating dense breast structures and smaller nodules. Thus, incorporating UE into BI-RADS could optimize the imaging evaluation system and offer a reliable basis for clinical decisions.

The BI-RADS classification stratifies BNs by morphological features, such as boundary, echogenicity, and vascularity. Nevertheless, a significant drawback is its failure to incorporate tissue stiffness, a crucial biological characteristic.^[12]^ UE complements this by quantitatively assessing nodular stiffness through parameters like shear wave velocity and strain rate. Such metrics are suggestive of cellular density and fibrotic alterations, with firmer tissues generally indicating malignancy due to dense cellularity and significant collagen deposition.^[13]^ In this research, the BI-RADS + UE combination, by integrating morphological and stiffness-related parameters, increased the accuracy of classifying benign and malignant BNs. Its advantages are particularly evident in the following 2 aspects: Reducing misdiagnosis and missed diagnosis: BI-RADS often incorrectly classifies nodules with well-defined boundaries but uneven internal echoes – such as fibroadenomas and atypical hyperplasia – as benign.^[14]^ UE, however, can help distinguish these nodules by detecting differences in stiffness. For instance, in this study, the higher true negative rate by the combined approach versus the BI-RADS-only strategy may be attributed to the ability of UE to specifically identify the stiffness of invasive carcinoma. Improving the identification of high-risk nodules: UE has low sensitivity to interfering factors such as microcalcification and inflammatory edema. BI-RADS, on the other hand, compensates for this deficiency by analyzing features like calcification morphology and blood flow signals.^[15]^ Similarly, when Zhu team explored the diagnostic effect of BI-RADS plus UE on BC, they arrived at the same conclusion as this study.^[16]^

In patients with radiologically dense breasts (ACR categories C/D), the abundant glandular structures may obscure nodule visualization on conventional ultrasound and thus elevate the risk of diagnostic oversights.^[17]^ This study found that the combined group also had a higher accuracy rate in diagnosing dense BNs than the BI-RADS group. This may be explained by the following mechanism: In dense glandular tissue, benign nodules – such as fibroadenomas – may exhibit morphological features similar to those of malignant nodules (e.g., ill-defined boundaries) due to compression by the surrounding glands. By detecting differences in stiffness, elastography enables effective distinguishing between these 2 types of nodules.^[18]^ For small nodules (≤10 mm), their small size and weak blood flow signals make them prone to being missed or misjudged in traditional ultrasound examinations.^[19]^ This study revealed that the combined protocol also achieved higher diagnostic accuracy for small nodules. Two factors may contribute to this outcome: First, UE exhibits high sensitivity in detecting small lesions. Shear wave elastography can penetrate up to 3cm into the tissue, making it feasible to measure the stiffness of nodules smaller than 5 mm in size. Second, UE overcomes certain limitations inherent to the BI-RADS system regarding morphological characterization. Small nodules often present with atypical structures, such as unclear boundaries and heterogeneous echogenicity, which often lead to a BI-RADS 4A classification. Elastography, by providing information about nodule stiffness, can mitigate the risk of over-classification.

Based on these findings, we propose incorporating the BI-RADS-UE combination into BN diagnostic guidelines, particularly for screening dense breast tissue and small lesions. Wider clinical implementation, however, requires unified operational criteria – including elastography compression depth and ROI selection – to decrease inter-observer discrepancies. Of course, this study also has several limitations that need to be addressed in future research. For example, this was a single-center study. Although the sample size met the statistical power requirements calculated by G-Power, multi-center, large-sample studies are still needed to verify the generalizability of the combined detection method. In addition, this study did not track long-term clinical outcomes, such as recurrence or metastasis. In the future, the follow-up period should be extended to evaluate the long-term predictive value of the combined protocol. Furthermore, MRI is known to provide better accuracy in identifying small lesions and evaluating dense breast tissue. Further studies may therefore be designed to evaluate the diagnostic potential of integrating BI-RADS, UE, and MRI.

In summary, integrating BI-RADS classification with UE enhances the differentiation between benign and malignant BNs by combining morphological and stiffness characteristics. This technique is especially advantageous in complex cases involving dense breasts or small lesions. It offers a more precise imaging tool for early BC screening and holds significant clinical potential. Future efforts should focus on expanding sample sizes, prolonging follow-up periods, and exploring the integration of multi-modal imaging technologies to advance precision medicine in BC diagnosis and treatment.

## Author contributions

**Conceptualization:** Lin Lu, Limin Zhu, Jing Li, Pei Zou.

**Data curation:** Lin Lu, Limin Zhu, Jing Li, Pei Zou.

**Formal analysis:** Lin Lu, Limin Zhu, Jing Li, Pei Zou.

**Funding acquisition:** Pei Zou.

**Investigation:** Lin Lu, Pei Zou.

**Writing** – **original draft:** Lin Lu, Limin Zhu, Jing Li, Pei Zou.

**Writing** – **review & editing:** Lin Lu, Limin Zhu, Jing Li, Pei Zou.
